# Does Political Trust Foster or Hinder Volunteering? A Longitudinal Investigation in the United Kingdom

**DOI:** 10.1177/19485506251411575

**Published:** 2026-01-08

**Authors:** Fanny Lalot, Dominic Abrams

**Affiliations:** 1 University of Basel, Switzerland; 2 University of Kent, Canterbury, UK

**Keywords:** civic engagement, institutional trust, political trust, volunteering, volunteers

## Abstract

Volunteering plays a growing role in supporting community resilience in times of crisis. This paper takes a closer look at the role of political trust for volunteering, distinguishing between trust in local and national government. We conduct secondary analysis of a large-scale longitudinal social survey in the United Kingdom (three timepoints, *N* = 5,039), to estimate a random-intercept cross-lagged panel model to assess the relationships between political trust and volunteering over time. Results reveal a positive link at the between-person level between volunteering and local but not national trust (random intercepts). At the within-person level (cross-lagged paths), an increase in local trust is related to a *greater* likelihood of volunteering 3 months later. Moreover, an increase in national trust is related to a *lower* likelihood of volunteering. We highlight implications for initiatives to promote civic engagement.

In the current era marked by multiple intersecting crises, communities are increasingly confronted with urgent and complex challenges. As these crises intensify, so too does the need for volunteers, whose contributions play a crucial role in addressing urgent societal needs and supporting community resilience (United Nations Volunteers, 2021).

A growing literature suggests that different degrees of political trust may either foster or hinder volunteering. The present paper draws from data collected during the COVID-19 pandemic to examine the temporal linkages between volunteering and trust in times of crisis. Extending past research, we focus not only on the degree of trust but also distinguish between two levels of governance: national and local political trust, which we suspect might influence volunteering in opposite directions, explaining mixed findings in the literature. On one hand, political trust may be negatively associated with volunteering (if trusting citizens believe institutions are already doing enough, or conversely if distrusting citizens organise themselves to make up for the absence of competent institutions)—an interpretation that seems especially plausible when focusing on trust in the *national* government. On the other hand, political trust may be positively associated with volunteering (when greater confidence in institutions, and more frequent contact with them, strengthens beliefs that personal investment is safe and worthwhile), which seems more likely when considering trust in *local* government. This study examines these possibilities.

## What Is Volunteering?

Omoto and Packard (2016) defined volunteering as:

an active and intentional process in which individuals and groups seek out opportunities to assist others. These actions, intended to be helpful, are undertaken by choice, on the basis of free will, and often in the service of personal values, needs, and motives. (p. 272)

People may engage in volunteering for different reasons that fulfil either more personal or more social motives (see Clary et al., 1998; Clary & Snyder, 1999). Volunteering typically benefits strangers, thus extending prosociality beyond close ties (Omoto & Packard, 2016; Snyder & Omoto, 2008). Early work emphasised *formal* volunteering, that is, through or on behalf of an organisation, while giving less attention to *informal* volunteering, that is, volunteering arranged or committed without institutional management (see Paine et al., 2010; Pearce & Kristjansson, 2019; Snyder & Omoto, 2008; Thomson, 2002). Informal volunteering includes actions such as mowing the lawn or running errands for an elderly neighbour, tutoring for free, picking up litter and so on. Recent estimates indicate that twice as many people volunteer informally as volunteer formally (United Nations Volunteers, 2021), highlighting the importance to consider both forms.

While volunteering is often considered a form of civic participation, it differs from other forms in several respects. Unlike much political participation or activism, volunteering is typically *not* intended to challenge or change the societal system. Rather, it accepts existing social structures and seeks to mitigate problems within this system without necessarily addressing root causes (Wong, 2024). It is also more often undertaken by members of wealthier or dominant groups, unlike typical minority-group-based collective action (Hustinx et al., 2022). Accordingly, volunteerism and civic action are believed to arise from complementary processes in the civil sphere (Janoski, 2010).

The literature identifies several personal antecedents of volunteering (Snyder & Omoto, 2008; Wilson, 2012), including personality traits (Bekkers, 2005; Omoto et al., 2010), empathic concern (Stürmer et al., 2005, 2006) and a stronger volunteer identity (Chacón et al., 2007; see also Grönlund, 2011). Volunteering can also be approached at the societal level, where the focus is on the linkages between individuals and the broader social structure (Snyder & Omoto, 2008). Identification with one’s community (Omoto & Packard, 2016) or neighbourhood (Johnson et al., 2018; Latham & Clarke, 2016; Pearce & Kristjansson, 2019) is related to greater volunteering.

Research at this level of explanation often adopts the lens of *social cohesion*, an umbrella term for the different vertical and horizontal interactions between individuals within a society (Bottoni, 2018a, 2018b; Chan et al., 2006). In general, volunteering is related to greater individual-level perceptions of social cohesion (Horsham et al., 2024; Lu et al., 2021; Niebuur et al., 2018). Places defined by higher levels of social cohesion also see greater rates of volunteering (Lalot et al., 2022); conversely, places marked by neighbourhood disorder (Latham & Clarke, 2016) or lower trust (Putnam, 2007) show lower rates.

The social cohesion literature also highlights the role of institutions for volunteering (most often under the form of perceived legitimacy of, and trust in institutions, contributing to *vertical cohesion*). This echoes the institutional theory of volunteerism, which argues that a ‘correct’ institutional context (including laws, policies and incentives) is necessary for the nonprofit sector to develop (Healy, 2004; Rotolo & Wilson, 2011; see also Snyder & Omoto, 2008). Countries with a longer and stable history of democratic regimes report higher rates of volunteering (Curtis et al., 2001), potentially through increased social trust. However, the temporal linkages between institutional (or political) trust and volunteering remain underexplored.

## The Role of Political Trust

Political trust refers to the confidence people have in their government (Levi & Stoker, 2000). It is thus an evaluative attitude reflecting citizens’ perceptions of the political system in several respects, often summarised as a government’s ability, benevolence and integrity (Devine et al., 2020; PytlikZillig & Kimbrough, 2016). Although unsurprisingly related to political partisanship (Hooghe & Oser, 2017), political trust cannot be reduced to a mere partisan bias. Partisans might grow dissatisfied with government performance or policy choices, and nonpartisans may still view government as competent and ethical despite disagreeing with certain policies (Citrin & Stoker, 2018; Hamm et al., 2019). Political trust influences citizen’s behaviour, including, for example, political engagement (Levi & Stoker, 2000). Here, we argue that it also matters for volunteering as a specific form of civic participation.

### Mixed Findings in the Literature

Our argument builds on the institutional theory of volunteerism, which highlights the role of adequate institutional incentives to maintain and develop the nonprofit sector (Rotolo & Wilson, 2011; see also Irwin, 2009). Accordingly, people should volunteer more when they trust that the general organisational structure is effective and non-corrupt, making their investment worthwhile and safe (Bolton, 2015; Wilson, 2000). Moreover, multicomponent models of social cohesion consider both political trust (vertical ties) and volunteering (horizontal ties) as indicators of social cohesion (Bottoni, 2018a, 2018b), implicitly suggesting that they should positively relate to one another.

This theoretical argument is plausible, but empirical evidence remains sparse and mixed.^1^ A recent review considered the relationships between volunteering and social cohesion as a whole but not with political trust specifically (Horsham et al., 2024; see also Lu et al., 2021). Taniguchi (2013) found a positive effect of trust in institutions (private and public) on irregular, but not regular, volunteering. Evers and Gesthuizen (2011) also found that people with higher political trust made larger donations to non-political organisations. However, Kohut (1998) observed no significant relationship between political trust and volunteering likelihood. Taniguchi and Marshall (2014) observed a positive link between political trust and charitable giving but not volunteering.

*Negative* relationships also appear, suggesting that citizens may organise horizontally to compensate distrusted institutions (Kaase, 1999). ‘Institutional trust is unlikely to increase volunteering when people are using their volunteer time to [. . .] work in some way to ameliorate the conditions created by a government they do not trust’ (Wilson, 2000, p. 225; see also Sivesind et al., 2013; van Ingen & van der Meer, 2016). Alternatively, citizens who believe that institutions function well may believe their own philanthropic or altruistic efforts are less necessary (as Evers & Gesthuizen, 2011, observed at the country level).

The question of causality also remains unanswered. Indeed, volunteering could also increase political trust: volunteering may bring people into contact with formal institutions, and this increased contact may increase trust (Bolton, 2015). As such, Fitzgerald and Wolak (2014) identified small but significant effects of voluntary organisation membership on political trust. Sivesind et al. (2013) notably found that volunteering increased political trust but only in a country with overall high levels of trust (i.e., in Norway but not the Czech Republic), which they argue highlights the different meanings of volunteering in (un)trustworthy institutional contexts.

### Trust in the National or Local Government

We propose that these mixed findings may be explained, at least partly, by the *level* at which political trust is conceived. Trust can refer to different political figures and institutions (e.g., local vs. state governance). Although ‘halo’ effects may produce consistent and intercorrelated evaluations of different political actors (Christensen & Lægreid, 2005; PytlikZillig et al., 2016), people also make distinctions. Trust in the local government is often higher than in the national government, potentially because the latter is more abstract (Abrams, Broadwood, Lalot, & Davies Hayon, 2021; Frederickson & Frederickson, 1995; Steenvoorden & van der Meer, 2021)—although not in every country (Fitzgerald & Wolak, 2014; Wu & Wilkes, 2017). Ability-based trust may also arise from different evaluations of government performance: national trust depends more on economic expectations, and local trust on positive evaluations of local performances and services (DeHoog et al., 1990; Steenvoorden & van der Meer, 2021).

We argue that both the *degree* and *direction* of the trust-volunteering link depend on the level at which trust is applied. Specifically, we might expect that political trust is negatively related to volunteering, either because citizens feel the institutions are doing enough or because they self-organise to compensate for a lack of trustworthy institutions, which seems most likely when considering trust in *national* government. Conversely, political trust could be positively related to volunteering because of increased contact with institutions and the belief that personal investment is both worthwhile and safe. This seems most likely when considering trust in *local* government. The present study aims to investigate these possibilities. We also explore the reverse relation, that is, from volunteering to trust in the national and local government.

## The Present Study

This paper aims to investigate what relationships may exist between volunteering and political trust, distinguishing between trust in the local versus national government. Our investigation relies on secondary analysis of a large-scale longitudinal social survey conducted in the United Kingdom during the COVID-19 pandemic, the Beyond Us and Them project (Abrams, Broadwood, Lalot, Davies Hayon, & Dixon, 2021). An eight-wave survey conducted from 2020 to 2021, it assessed topics related to social cohesion, including (relevant for the present purpose) political trust and volunteering.

### Transparency and Openness

Data, materials and code for analysis are publicly available on the OSF: https://osf.io/e3anb. All analyses were conducted on RStudio 2023.06.0+421 using packages *lavaan* (Rosseel, 2012), *ChiBarSq.DiffTest* (Kuiper, 2025), and *powRICLPM* (Mulder, 2023). This secondary data analysis was not preregistered.

### Method

#### Participants

We focus on longitudinal data from three consecutive waves (conducted in December 2020, March 2021, and June 2021) as these included a consistent set of measures of political trust and volunteering and provided a large longitudinal sample of *N* = 5,039 (of which 2,407 completed two consecutive waves only, and 2,632 completed all three waves). The sample included 2,216 men (44.0%), 2,805 women (55.7%) and 18 ‘other’ or undisclosed (0.3%), of a mean age of 47.15 (*SD* = 16.59). A full demographic profile, including a comparison between participants returning and not returning to a subsequent wave, is reported in Supplementary Material (SM1).

#### Materials

##### Political Trust at the National Versus Local Level

Our methodology was necessarily shaped by the measures available in the dataset. Given our aim to contrast trust in the national versus local government, we could only utilise a single-item measure for each. These items focused on trust in the government response to COVID-19: ‘I believe [the United Kingdom Government / my local council (i.e., town)] is handling the causes and consequences of the pandemic competently’ (5-point Likert-type scale, 1 = *strongly disagree*, 5 = *strongly agree*) and were included in each wave. They arguably capture trust primarily through the lens of government’s perceived *ability* (Davies et al., 2021)—an important component of political trust (Hamm et al., 2019). Trust in the government response to COVID-19 was surveyed closely in several large-scale surveys during the pandemic, including the COVIDistress Survey (Yamada et al., 2021), the University of Leeds and Savanta ComRes Covid-19 Messaging Project (Coleman et al., 2020), and the Imperial College London YouGov Covid 19 Behaviour Tracker (Institute of Global Health Innovation, 2022), with similarly worded items (see Davies et al., 2021, for an overview).

As this research and others indicate, the pandemic affected virtually every domain of public and private life, including close relationships and well-being, jobs and economic growth, education, arts and culture, prejudice and discrimination. Thus, it can be argued that the items assessing how the government handled the ‘causes and consequences’ of the pandemic tap into the perception of society broadly speaking across a range of domains.

##### Volunteering

Volunteering was retrieved from a larger checklist that included different forms of political and civic action (e.g., signed a petition, made a donation). Participants indicated in a dichotomous format (0 = *no*, 1 = *yes*) whether they had engaged in the activity during the past month. In the first wave, a single item assessed ‘Volunteered’. In waves 2 and 3, two items separately captured ‘Volunteered “formally” (through an official charity or organisation)’ and ‘Helping out “informally” (personally or through an informal organisation such as a mutual aid group)’. To ensure comparability across waves, we created a single score from these two questions, coded 0 = *Engaged in neither*, 1 = *Engaged in at least one form of volunteering*.

Volunteering is conceptually distinct from other forms of political and civic action, but we also checked to ensure that this was the case empirically. When we conducted exploratory factor analyses on the civic participation items, at every wave, the items for volunteering (and donating) loaded on a different factor than all others, thus supporting our choice to focus on volunteering specifically (see SM2). As a robustness check, we also investigated the relationships between trust and *donating* (single item) as well as a single indicator of donating or volunteering. These tests yielded similar results to our main analysis (see SM3). Descriptive statistics are reported in Table 1.

**Table 1:** Descriptive Statistics of Political Trust and Volunteering at Each Measurement Time

Variable	Time 1	Time 2	Time 3
Trust in national government *M* (*SD*)	2.28 (1.23)	2.58 (1.28)	2.55 (1.24)
Trust in local government *M* (*SD*)	3.12 (1.05)	3.24 (1.03)	3.20 (1.00)
Volunteering (% yes)	16.3%	12.8%	12.6%

*Note*. Trust is measured on a 5-point Likert-type scale; volunteering is a dichotomous yes/no measure.

#### Analysis Strategy

We relied on a random intercept cross-lagged panel model approach (RI-CLPM) to investigate the longitudinal relations between political trust (at the national and local level) and volunteering. The RI-CLPM separates stable, between-person differences from dynamic, within-person processes, enabling us to disentangle long-term trait-like associations from time-specific state-like interactions (through both autoregressive and cross-lagged effects). This approach improves causal inference and avoids conflating between-person and within-person effects common in traditional CLPMs (Hamaker, 2023; Hamaker et al., 2015).

**Figure 1:**
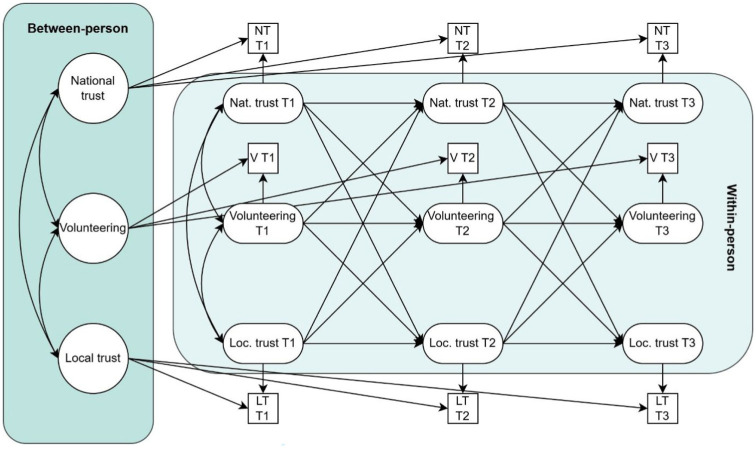
Representation of the Random-Intercept Cross-Lagged Model Tested in the Study *Note.* Although not depicted for simplification purposes, we also estimated the covariances between residuals of the within-person centred variables.

We estimated an RI-CLPM where trust in the national government, trust in the local government, and volunteering at Time 1 and Time 2 were used to predict each other at Time 2 and Time 3, respectively (see Figure 1). For each construct, we computed a between-person latent variable (or random intercept) as well as single indicator latent variables at each time point, which capture within-person effects (i.e., time-specific deviations from each participant’s trait-level means). We calculated covariances between the between-person latent indicators and all autoregressive and cross-lagged paths between within-person time-specific indicators. As we had no theoretical reasons to predict that the strength of these associations would vary through time (i.e., between Time1–Time2 and Time2–Time3), we constrained each pair of (autoregressive and cross-lagged) associations to be equal across time. Complementary analysis showed that imposing these constraints only minimally reduced model fit, Δχ^2^(Δdf = 9) = 31.46, *p* < .001, ΔCFI = .002, ΔRMSEA = .009, ΔSRMR = .006, ΔGFI < .001, reinforcing our choice to rely on the more parsimonious model.

To account for the dichotomous nature of the volunteering measure, we relied on the WLSMV estimator with theta parametrisation, which uses diagonally weighted least squares (DWLS) to estimate the model parameters, but uses the full weight matrix to compute robust standard errors, and a mean- and variance-adjusted test statistic.^2^ It should be noted that for binary outcomes (here, volunteering), standardised coefficients are defined on the underlying latent response variable and should *not* be interpreted directly as standardised effects on the observed binary variable. We thus additionally calculated the marginal effects of trust on volunteering, or the percentage-point change in P(volunteering = 1) per 1-unit increase in the predictor, at the within-person mean or η = 0.

A power analysis (1,500 simulations) indicated that our sample size provided >.95 power to detect a standardised cross-lagged effect as small as .10 (three time points, other indicators set conservatively to small values: ICC = .40, RI correlation = .20, autoregressive effects = .20, opposite cross-lagged effect = .07; Mulder, 2023).

### Results

**Figure 2:**
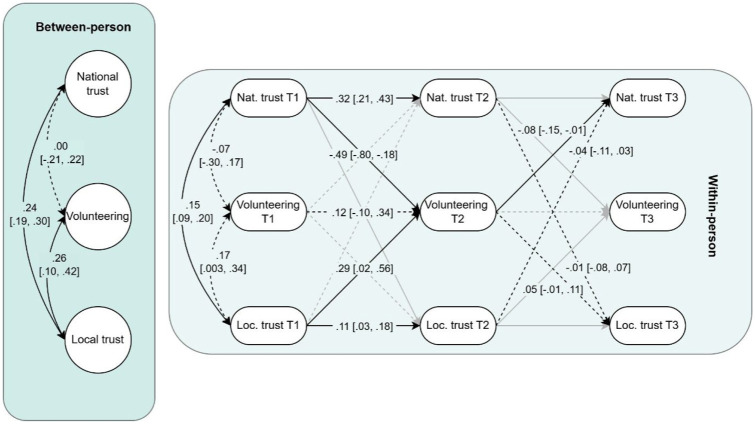
Random-Intercept Cross-Lagged Model Testing the Associations Between Trust in the National and Local Government and Volunteering *Note.* The numbers reported are unstandardised coefficients with 95% CI. As the autoregressive and cross-lagged coefficients were fixed to be equal from T1-T2 and from T2-T3, we report them only once for visual simplification purposes.

Results are depicted in Figure 2, and the full output is reported in Table 2. The RI-CLPM provided excellent fit to the data, χ^2^ = 173.86, df = 22, χ^2^/df = 7.90, CFI = .983, RMSEA = .037, 90% CI [.032, .042], SRMR = .016.

**Table 2:** Results From the Random-Intercept Cross-Lagged Model Testing the Associations Between Trust in the National and Local Government and Volunteering

Association	Est.	*SE*	95% CI	*z*-test	*p*-value	β (T1 / T2)
**Between-person covariances (random intercepts)**						
National trust ∼∼ Local trust	.244	.028	[.189, .299]	8.64	<.001	.306
National trust ∼∼ Volunteering	.003	.109	[−.210, .216]	0.03	.98	.001
Local trust ∼∼ Volunteering	.261	.080	[.104, .418]	3.26	.001	.151
**Within-person covariances at T1**						
National trust ∼∼ Local trust	.147	.027	[.094, .199]	5.49	<.001	.299
National trust ∼∼ Volunteering	−.068	.119	[−.301, .165]	-0.57	.57	−.061
Local trust ∼∼ Volunteering	.169	.085	[.003, .335]	1.99	.046	.148
**Within-person covariances at T2 & T3**						
National trust ∼∼ Local Trust	.128	.016	[.096, .160]	7.89	<.001	.289
National trust ∼∼ Volunteering	−.137	.056	[−.248, .027]	-2.43	.015	−.199
Local trust ∼∼ Volunteering	−.055	.049	[−.151, .042]	-1.12	.27	−.086
**Within-person regressions at T2 & T3**						
National trust_T_						
∼ National trust_T-1_	.319	.055	[.211, .428]	5.77	<.001	.301 / .318
∼ Local trust_T-1_	−.043	.035	[−.112, .025]	-1.24	.22	−.042 / −.038
∼ Volunteering_T-1_	−.079	.035	[−.148, −.011]	-2.27	.024	−.173 / −.116
Local trust_T_						
∼ National trust_T-1_	−.005	.040	[−.083, .073]	-0.12	.90	−.005 / −.006
∼ Local trust_T-1_	.109	.038	[.034, .184]	2.84	.005	.118 / .110
∼ Volunteering_T-1_	.053	.031	[−.008, .114]	1.71	.088	.131 / .089
Volunteering_T_						
∼ National trust_T-1_	−.487	.157	[−.795, −.180]	-3.11	.002	−.312 / −.331
∼ Local trust_T-1_	.290	.138	[.019, .561]	2.09	.036	.189 / .175
∼ Volunteering_T-1_	.120	.110	[−.096, .336]	1.09	.28	.178 / .120

*Note*. Covariances are indicated as x_1_∼∼ x_2_. Regressions are indicated as y ∼ x (i.e., the fully left-justified variable is the endogenous variable). Within-person covariances at Time 1 reflect the relationship between the variables after accounting for their stable, trait-like individual differences, but not influenced by prior time points (since it is the first measurement). Within-person covariances at Times 2-3 represent the residual (state-like) associations, controlling for both stable, trait-like differences (random intercepts) and previous within-person fluctuations due to the cross-lagged and autoregressive processes. It should be noted that for binary outcomes (here, volunteering), standardised coefficients are defined on the underlying latent response variable and should not be interpreted directly as standardised effects on the observed binary variable. Although the point estimate is constrained to equality, the effects are standardised based on the point estimate and variances, and since the variances vary at each assessment occasion, the standardised effects are slightly different at Time 2 and Time 3.

#### Between-Person Level: Random Intercepts

At the between-person level, the random intercepts of trust in the national and the local government were positively correlated (β = .31, *p* < .001), indicating that participants who reported higher national trust overall also reported higher local trust. The random intercepts of local trust and volunteering were also significantly related (β = .15, *p* < .001): participants who reported higher local trust overall were also more likely to volunteer. The random intercepts of national trust and volunteering, on the other hand, were not significantly related (β = .001, *p* = .98). As a robustness check, we repeated the analysis while controlling for demographics. This yielded virtually identical results.^3^ More detail regarding the role of demographics on trust and volunteering is reported in SM4.

#### Within-Person Level: Regressions

##### Auto-Regressive Paths

Results showed significant autoregressive paths for national trust (β_T1/T2_ = .30/.32, *p* < .001) and local trust (β_T1/T2_ = .12/.11, *p* = .005), indicating that within-person fluctuations persist over time. This means that if an individual experienced a temporary increase (or decrease) in trust at one time point, they were more likely to maintain that deviation at the next time point. In contrast, the autoregressive path for volunteering was not significant (β_T1/T2_ = .18/.12, *p* = .28), suggesting volunteering fluctuated independently across time rather than following a stable pattern of inertia. In other words, volunteering behaviour appears to be rather situational, and fluctuations may not persist over time.

##### Cross-Lagged Paths

Finally, we turned to the cross-lagged paths. National trust at Time T was negatively predicted by volunteering (β_T1/T2_ = −.17/−.12, *p* = .024) at Time T-1, but not by local trust (β_T1/T2_ = −.04, *p* = .22). Local trust_T_ on the other hand was not significantly predicted by either national trust_T-1_ (β_T1/T2_ = −.01, *p* = .90) or volunteering_T-1_ (β_T1/T2_ = .13/.09, *p* = .088).

Finally, and most relevant to our research question, volunteering at Time T was significantly predicted by national trust and local trust at Time T-1 but in opposite directions. Specifically, a person who experienced a decrease in national trust would later be more likely to volunteer (β_T1/T2_ = −.31/−.33, *p* = .002; marginal effect = −.101 or decrease of 10.1 percentage points by 1-unit increase in national trust); and a person who experienced an increase in local trust would later be more likely to volunteer (β_T1/T2_ = .19/.18, *p* = .036; marginal effect = .060). Conversely, someone who experienced an increase in national trust and/or a decrease in local trust would become less likely to volunteer 3 months later.

## Discussion

The latest U.S. Census Bureau report indicates that, after a pandemic-era drop, volunteering is on the rise again (Hanson Schlachter & Marshall, 2024). Elsewhere, however, long-term trends point downwards. In the United Kingdom, both formal and informal volunteering have been declining for the past 10 years (the United Kingdom Department for Culture, Media & Sport, 2024). These figures and others worldwide (United Nations Volunteers, 2021) suggest that global crises may disrupt volunteerism with lasting repercussions for societal resilience to the extent that it may rely on informal social sources of mutual support. This highlights the need to identify factors that encourage volunteering and that could leverage faster recovery from a crisis.

The literature has yielded mixed findings regarding the links between political trust and volunteering. We present a longitudinal investigation of these relations, which highlights the importance of distinguishing between different levels of governance for understanding both the direction and strength of relationships between trust and volunteering.

### From Political Trust to Volunteering

We find opposite associations by level of trust. Local trust shows both synchronous and cross-lagged *positive* relations with volunteering: people with higher local trust are more likely to volunteer at a given point in time; moreover, experiencing an increase in local trust makes one more likely to volunteer 3 months later. National trust, on the other hand, only shows a cross-lagged *negative* relation: experiencing an increase in national trust makes one *less* likely to volunteer 3 months later.

These results clarify prior ambiguities in the literature, which had identified positive (e.g., Taniguchi, 2013), negative (e.g., Kaase, 1999), or no link (e.g., Kohut, 1998) between political trust and volunteering. Authors often utilised aggregate scores that average trust in different political institutions or actors, which, we argue, creates noise in the findings. We show here that the level of governance is key: trust in the local versus national government exert directionally opposite influences on volunteering that are of similar size. Had we not distinguished the two measures, their effects might have cancelled one another out.

### From Volunteering to Political Trust

We found a small negative cross-lagged effect of volunteering on trust in the national government. The effect on trust in the local government was directionally positive but did not reach significance (*p* = .088). In our robustness check, considering an index of donating or volunteering, however, the effect became significant (*p* = .008). Thus, rather than contradicting earlier findings, our evidence suggests that such effects may be small. Directionally, the effects of volunteering on trust are consistent with the opposing tendencies: positive effects on local trust and negative effects on national trust. The relatively small effects could reflect the time lag between waves. An algebraic calculation using our coefficients indicates that the ‘optimal time lag’ may have been shorter (1.5–2 months instead of 3; Dormann & Griffin, 2015), in part due to the low stability of the volunteering measure.^4^ Further studies on shorter time lags would be useful to advance our understanding of volunteering-trust dynamics. Indeed, time lags that are too long may lead to underestimating causal effects.

We must also consider the possibility that different dynamics may emerge over different time frames. Debate persists over the stability of political trust: some argue it crystallises in adolescence on the basis of early-life experiences, and thereafter shows only temporal fluctuations around a mean level (e.g., Devine & Valgarðsson, 2024; Krosnick & Alwin, 1989). Others contend civic and political experiences in adulthood, including volunteering, continue to durably shape trust (Sivesind et al., 2013). Our data can only speak to short-term fluctuations. Long-term investigations are needed to determine whether (sustained) volunteering may influence trust in the long run.

### Limitations and Future Directions

This study has several strengths, including a large longitudinal sample and state-of-the-art statistical analysis that separates synchronous and cross-lagged relations. However, some limitations must be acknowledged, which open avenues for future research.

First, our data were from a single nation (the United Kingdom). Different national contexts may produce different trust dynamics, depending, for example, on the average level of political trust (Evers & Gesthuizen, 2011; Sivesind et al., 2013) or collectivist-individualist culture (Taniguchi, 2013; Taniguchi & Marshall, 2014; Wu & Wilkes, 2017). Future work should investigate the effects of national versus local political trust across countries.

Second, evidence in the United Kingdom suggests that in times of crisis such as the COVID-19 pandemic, political trust is especially labile (e.g., Davies et al., 2021), and volunteering is especially crucial, making the present research particularly timely but possibly atypical. Crises lacking a ‘common fate’ narrative may yield different patterns, and it will be important to assess whether the dynamics revealed in our analyses persist in less turbulent times. Our trust measure was also context-specific as it assessed competence-based trust (adequacy of government actions to tackle the pandemic). While competence is a central component of trust (Hamm et al., 2019) and similar measures were widely used in pandemic surveys (Davies et al., 2021), future work should disentangle different components of trust (PytlikZillig et al., 2016).

Relatedly, the civic engagement literature tends to contrast low vs. high levels of trust; yet, low trust is not necessarily equivalent to *distrust*. This distinction remains a contention with different theoretical perspectives holding either that trust and distrust form a two-pole continuum (with mistrust being the neutral middle-point; Sitkin & Bijlsma-Frankema, 2018; Ullmann-Margalit, 2004), or that trust and distrust are distinct constructs leading to different, specific responses (Bertsou, 2019; Lewicki et al., 1998; Six & Latusek, 2023; see also Fletcher et al., 2024). Psychometrically, our scales align with the former: the lower end likely captures distrust and not merely low trust. Refining measurement would clarify the distinct dynamics of (low) trust, distrust and mistrust.

More specific forms of volunteering could also be considered. In line with much of the existing research, we focused here on volunteering occurrence, that is, whether people volunteered or not. But volunteering intensity and type should also be considered (see Horsham et al., 2024). Recent figures in the United States suggest that although the share of citizens who are volunteering is on the rise, the number of hours served per volunteer is decreasing (Hanson, Schlachter & Marshall, 2024). Future studies should look at such episodic volunteering and contrast its dynamics with that of sustained volunteering. Similarly, it would be interesting to separately consider the different motives for volunteering (Clary et al., 1998; Clary & Snyder, 1999). The impact of trust could be especially relevant for prosocial volunteering and maybe less so for self-oriented volunteering—something future work could assess.

#### What Underpins the Effect of Local Trust?

We cannot say for certain *what* it is exactly about trust in local government that facilitates volunteering. Citizens interact more directly with local government and see its impact on their daily life (i.e., a more concrete perception; Frederickson & Frederickson, 1995), which can make civic engagement feel more relevant. Indeed, an important component of volunteering (especially informal) occurs at the local level. We conjecture that trust may thus foster a belief that volunteering efforts will be well-utilised and lead to tangible results. It is also plausible that a trustworthy local government may be more likely to provide structured opportunities for participation, making it easier for residents to get involved. It may also allocate resources efficiently, reassuring people that their contribution will be meaningful. Local trust could thus contribute to a greater sense of self- and collective efficacy, as well as reinforce a sense of social cohesion within the community. Future research is needed to explore and pinpoint whether such mechanisms (i.e., concrete evaluation, greater contact, local engagement, perceived efficacy) underlie the positive effect of local trust on volunteering.

### Practical Implications and Conclusions

This research contributes to the literature on political trust as a potential antecedent of volunteering. It highlights the importance of considering different levels of governance separately and suggests that trust in the national and local government may contribute to opposing dynamics—national trust hindering and local trust encouraging volunteering.

A practical implication of these findings is that the most substantial gains in efforts to sustain civic engagement and volunteering efforts may be derived by focussing on local levels and building trust in local governance. This can be achieved through clearer and more honest communication (Abrams, Broadwood, Lalot, & Davies Hayon, 2021) and demonstrating consistent positive impact for the local community (Fitzgerald & Wolak, 2014). Such efforts may be even more important in times of crisis, where political trust is particularly fragile, and citizens may turn to their community (Aassve et al., 2024) and local level of government (Lalot et al., 2022) to look for guidance and organise their efforts. As such, we join others in calling for national institutions to work with local governments to support place-based approaches that put social cohesion at the heart of levelling up (Broadwood et al., 2021).

## Supplemental Material

sj-docx-1-spp-10.1177_19485506251411575 – Supplemental material for Does Political Trust Foster or Hinder Volunteering? A Longitudinal Investigation in the United Kingdom Supplemental material, sj-docx-1-spp-10.1177_19485506251411575 for Does Political Trust Foster or Hinder Volunteering? A Longitudinal Investigation in the United Kingdom by Fanny Lalot and Dominic Abrams in Social Psychological and Personality Science

## Notes

1. In contrast, a large literature has investigated relationships between volunteering and social trust and generally identifies positive and bidirectional effects, although some contention about directionality remains (e.g., Bekkers, 2012; Kaase, 1999; Putnam, 1993, 2007; Sivesind et al., 2013; Sønderskov, 2011; Wollebæk & Strømsnes, 2007).

2. See: https://lavaan.ugent.be/tutorial/cat.html.

3. When controlling for demographics (age, gender, political orientation, ethnicity and subjective socioeconomic status), the random intercepts of trust in the national and the local government were positively correlated (β = .36, *p* < .001), and so were the random intercepts of local trust and volunteering (β = .18, *p* = .002). The random intercepts of national trust and volunteering were not significantly related (β = −.01, *p* = .81).

4. We applied Equation 13 from Dormann and Griffin (2015) to the autoregressive and cross-lagged relationships between one type of trust and volunteering. For local government trust, this indicated an optimal time lag of 0.46 × the specific unit (SU) of time (i.e., 0.46 × 3 months = 1.37 months). For national government trust, this indicated an optimal time lag of 0.63 × SU (i.e., 0.63 × 3 months = 1.90 months).

## References

[bibr1-19485506251411575] Aassve A. Capezzone T. Cavalli N. Conzo P. Peng C. (2024). Social and political trust diverge during a crisis. Scientific Reports, 14(1), 331. 10.1038/s41598-023-50898-4 38172518 PMC10764309

[bibr2-19485506251411575] Abrams D. Broadwood J. Lalot F. Davies Hayon K. (2021, August 17). People largely perceive local government communications about COVID-19 as embodying greater honesty, credibility, and empathy than those of the UK government. LSE British Politics and Policy. https://blogs.lse.ac.uk/politicsandpolicy/government-communication-covid19/

[bibr3-19485506251411575] Abrams D. Broadwood J. Lalot F. Davies Hayon K. Dixon A. (2021). Beyond us and them: Societal cohesion in Britain through eighteen months of COVID-19. Belong – The Cohesion and Integration Network. https://www.belongnetwork.co.uk/resources/beyond-us-and-them-societal-cohesion-in-britain-through-eighteen-months-of-covid-19/

[bibr4-19485506251411575] Bekkers R. (2005). Participation in voluntary associations: Relations with resources, personality, and political values. Political Psychology, 26(3), 439–454. 10.1111/j.1467-9221.2005.00425.x

[bibr5-19485506251411575] Bekkers R. (2012). Trust and volunteering: Selection or causation? Evidence from a 4 year panel study. Political Behavior, 34(2), 225–247. 10.1007/s11109-011-9165-x

[bibr6-19485506251411575] Bertsou E. (2019). Rethinking political distrust. European Political Science Review, 11(2), 213–230. 10.1017/S1755773919000080

[bibr7-19485506251411575] Bolton V. (2015). Volunteering and political engagement: An empirical investigation [PhD thesis]. University of Southampton.

[bibr8-19485506251411575] Bottoni G. (2018a). A multilevel measurement model of social cohesion. Social Indicators Research, 136(3), 835–857. 10.1007/s11205-016-1470-7

[bibr9-19485506251411575] Bottoni G. (2018b). Validation of a social cohesion theoretical framework: A multiple group SEM strategy. Quality & Quantity, 52(3), 1081–1102. 10.1007/s11135-017-0505-8

[bibr10-19485506251411575] Broadwood J. Lalot F. Abrams D. Davies Hayon K. (2021, May 24). The government must work with local government to support a place-based approach that puts social cohesion at the heart of levelling up. LSE British Politics and Policy. https://blogs.lse.ac.uk/politicsandpolicy/social-cohesion-covid19/

[bibr11-19485506251411575] Chacón F. Vecina M. L. Dávila M. C. (2007). The three-stage model of volunteers’ duration of service. Social Behavior and Personality, 35, 627–642. 10.2224/sbp.2007.35.5.627

[bibr12-19485506251411575] Chan J. To H.-P. Chan E. (2006). Reconsidering social cohesion: Developing a definition and analytical framework for empirical research. Social Indicators Research, 75(2), 273–302. 10.1007/s11205-005-2118-1

[bibr13-19485506251411575] Christensen T. Lægreid P. (2005). Trust in government: The relative importance of service satisfaction, political factors, and demography. Public Performance & Management Review, 28(4), 487–511. 10.1080/15309576.2005.11051848

[bibr14-19485506251411575] Citrin J. Stoker L. (2018). Political trust in a cynical age. Annual Review of Political Science, 21(1), 49–70. 10.1146/annurev-polisci-050316-092550

[bibr15-19485506251411575] Clary E. G. Snyder M. (1999). The motivations to volunteer: Theoretical and practical considerations. Current Directions in Psychological Science, 8(5), 156–159. 10.1111/1467-8721.00037

[bibr16-19485506251411575] Clary E. G. Snyder M. Ridge R. D. Copeland J. Stukas A. A. Haugen J. Miene P. (1998). Understanding and assessing the motivations of volunteers: A functional approach. Journal of Personality and Social Psychology, 74(6), 1516–1530. 10.1037/0022-3514.74.6.1516 9654757

[bibr17-19485506251411575] Coleman S. Konstantinova N. Moss G. (2020). The pandemic and its publics – How people receive, interpret and act upon official guidance. https://ahc.leeds.ac.uk/media/doc/communicating-pandemic

[bibr18-19485506251411575] Curtis J. E. Baer D. E. Grabb E. G. (2001). Nations of joiners: Explaining voluntary association membership in democratic societies. American Sociological Review, 66(6), 783–805. 10.1177/000312240106600601

[bibr19-19485506251411575] Davies B. Lalot F. Peitz L. Heering M. S. Ozkececi H. Babaian J. Davies Hayon K. Broadwood J. Abrams D. (2021). Changes in political trust in Britain during the COVID-19 pandemic in 2020: Integrated public opinion evidence and implications. Humanities and Social Sciences Communications, 8, 166. 10.1057/s41599-021-00850-6

[bibr20-19485506251411575] DeHoog R. H. Lowery D. Lyons W. E. (1990). Citizen satisfaction with local governance: A test of individual, jurisdictional, and city-specific explanations. The Journal of Politics, 52(3), 807–837. 10.2307/2131828

[bibr21-19485506251411575] Devine D. Gaskell J. Jennings W. Stoker G. (2020). Exploring trust, mistrust and distrust [Discussion paper]. 1st Digital Workshop of the ESRC ‘TrustGov’ Project. https://static1.squarespace.com/static/5c21090f8f5130d0f2e4dc24/t/5e995ec3f866cd1282cf57b1/1587109577707/TrustGov+-+Trust+mistrust+distrust+-+20.04.2020.pdf

[bibr22-19485506251411575] Devine D. Valgarðsson V. O. (2024). Stability and change in political trust: Evidence and implications from six panel studies. European Journal of Political Research, 63(2), 478–497. 10.1111/1475-6765.12606

[bibr23-19485506251411575] Dormann C. Griffin M. A. (2015). Optimal time lags in panel studies. Psychological Methods, 20(4), 489–505. 10.1037/met0000041 26322999

[bibr24-19485506251411575] Evers A. Gesthuizen M. (2011). The impact of generalized and institutional trust on donating to activist, leisure, and interest organizations: Individual and contextual effects. International Journal of Nonprofit and Voluntary Sector Marketing, 16(4), 381–392. 10.1002/nvsm.434

[bibr25-19485506251411575] Fitzgerald J. Wolak J. (2014). The roots of trust in local government in western Europe. International Political Science Review, 37(1), 130–146. 10.1177/0192512114545119

[bibr26-19485506251411575] Fletcher K. A. Summers J. K. Bedwell-Torres W. L. Humphrey S. E. Thomas S. E. Ramsay P. S. (2024). Initial development of perceptions of ability and intent factors of (un)trustworthiness in short-term teams. Journal of Organizational Behavior, 45(7), 1025–1046. 10.1002/job.2795

[bibr27-19485506251411575] Frederickson H. G. Frederickson D. G. (1995). Public perceptions of ethics in government. The ANNALS of the American Academy of Political and Social Science, 537(1), 163–172. 10.1177/0002716295537000014

[bibr28-19485506251411575] Grönlund H. (2011). Identity and volunteering intertwined: Reflections on the values of young adults. VOLUNTAS: International Journal of Voluntary and Nonprofit Organizations, 22(4), 852–874. 10.1007/s11266-011-9184-6

[bibr29-19485506251411575] Hamaker E. L. (2023). The within-between dispute in cross-lagged panel research and how to move forward. Psychological Methods. Advance online publication. 10.1037/met0000600 37902677

[bibr30-19485506251411575] Hamaker E. L. Kuiper R. M. Grasman R. P. P. P. (2015). A critique of the cross-lagged panel model. Psychological Methods, 20(1), 102–116. 10.1037/a0038889 25822208

[bibr31-19485506251411575] Hamm J. A. Smidt C. Mayer R. C. (2019). Understanding the psychological nature and mechanisms of political trust. PLOS ONE, 14(5), Article e0215835. 10.1371/journal.pone.0215835 PMC6519795 31091243

[bibr32-19485506251411575] Hanson Schlachter L. Marshall T. (2024). U.S. volunteerism rebounding after COVID-19 pandemic. https://www.census.gov/library/stories/2024/11/civic-engagement-and-volunteerism.html

[bibr33-19485506251411575] Healy K. (2004). Altruism as an organizational problem: The case of organ procurement. American Sociological Review, 69(3), 387–404. 10.1177/000312240406900304

[bibr34-19485506251411575] Hooghe M. Oser J. (2017). Partisan strength, political trust and generalized trust in the United States: An analysis of the General Social Survey, 1972–2014. Social Science Research, 68, 132–146. 10.1016/j.ssresearch.2017.08.005 29108592

[bibr35-19485506251411575] Horsham Z. Abrams D. Davies B. Lalot F. (2024). Social cohesion and volunteering: Correlates, causes, and challenges. Translational Issues in Psychological Science, 10(1), 51–68. 10.1037/tps0000387

[bibr36-19485506251411575] Hustinx L. Grubb A. Rameder P. Shachar I. Y. (2022). Inequality in volunteering: Building a new research front. Voluntas, 33(1), 1–17. 10.1007/s11266-022-00455-w 35095218 PMC8791087

[bibr37-19485506251411575] Institute of Global Health Innovation. (2022). COVID-19 global behaviours and attitudes: A review of the survey results of over 450,000 people in 9 countries. https://www.imperial.ac.uk/media/imperial-college/institute-of-global-health-innovation/Two-year_ICL-YouGov-Covid-19-Behaviour-Tracker-FINAL.pdf

[bibr38-19485506251411575] Irwin K. (2009). Prosocial behavior across cultures: The effects of institutional versus generalized trust. In Thye S. R. Lawler E. J. (Eds.), Altruism and prosocial behavior in groups (Vol. 26, pp. 165–198). Emerald Group Publishing Limited. 10.1108/S0882-6145(2009)0000026010

[bibr39-19485506251411575] Janoski T. (2010). The dynamic processes of volunteering in civil society: A group and multi-level approach. Journal of Civil Society, 6(2), 99–118. 10.1080/17448689.2010.506367

[bibr40-19485506251411575] Johnson K. J. Latham-Mintus K. Poey J. L. (2018). Productive aging via volunteering: Does social cohesion influence level of engagement? Journal of Gerontological Social Work, 61(8), 817–833. 10.1080/01634372.2018.1467523 29697314

[bibr41-19485506251411575] Kaase M. (1999). Interpersonal trust, political trust and non-institutionalised political participation in Western Europe. West European Politics, 22(3), 1–21. 10.1080/01402389908425313

[bibr42-19485506251411575] Kohut A. (1998). Trust and citizen engagement in metropolitan Philadelphia: A case study. Pew Research Center for The People & The Press.

[bibr43-19485506251411575] Krosnick J. A. Alwin D. F. (1989). Aging and susceptibility to attitude change. Journal of Personality and Social Psychology, 57(3), 416–425. 10.1037/0022-3514.57.3.416 2778632

[bibr44-19485506251411575] Kuiper R. M. (2025). Chi-bar-square difference test of the RI-CLPM versus the CLPM and more general (Version 0.0.0.9000). https://github.com/rebeccakuiper/ChiBarSq.DiffTest

[bibr45-19485506251411575] Lalot F. Abrams D. Broadwood J. Davies Hayon K. Platts-Dunn I. (2022). The social cohesion investment: Communities that invested in integration programmes are showing greater social cohesion in the midst of the COVID-19 pandemic. Journal of Community & Applied Social Psychology, 32(3), 536–554. 10.1002/casp.2522 34230795 PMC8251431

[bibr46-19485506251411575] Latham K. Clarke P. J. (2016). Neighborhood disorder, perceived social cohesion, and social participation among older Americans: Findings from the National Health & Aging Trends Study. Journal of Aging and Health, 30(1), 3–26. 10.1177/0898264316665933 27566464

[bibr47-19485506251411575] Levi M. Stoker L. (2000). Political trust and trustworthiness. Annual Review of Political Science, 3(1), 475–507. 10.1146/annurev.polisci.3.1.475

[bibr48-19485506251411575] Lewicki R. J. McAllister D. J. Bies R. J. (1998). Trust and distrust: New relationships and realities. The Academy of Management Review, 23(3), 438–458. 10.2307/259288

[bibr49-19485506251411575] Lu P. Xu C. Shelley M. (2021). A state-of-the-art review of the socio-ecological correlates of volunteerism among older adults. Ageing and Society, 41(8), 1833–1857. 10.1017/S0144686X20000082

[bibr50-19485506251411575] Mulder J. D. (2023). Power analysis for the Random Intercept Cross-Lagged Panel Model using the powRICLPM R-package. Structural Equation Modeling: A Multidisciplinary Journal, 30(4), 645–658. 10.1080/10705511.2022.2122467 PMC7615284 37937063

[bibr51-19485506251411575] Niebuur J. van Lente L. Liefbroer A. C. Steverink N. Smidt N. (2018). Determinants of participation in voluntary work: A systematic review and meta-analysis of longitudinal cohort studies. BMC Public Health, 18(1), Article 1213. 10.1186/s12889-018-6077-2 PMC6214171 30384837

[bibr52-19485506251411575] Omoto A. M. Packard C. D. (2016). The power of connections: Psychological sense of community as a predictor of volunteerism. The Journal of Social Psychology, 156(3), 272–290. 10.1080/00224545.2015.1105777 27064179

[bibr53-19485506251411575] Omoto A. M. Snyder M. Hackett J. D. (2010). Personality and motivational antecedents of activism and civic engagement. Journal of Personality, 78(6), 1703–1734. 10.1111/j.1467-6494.2010.00667.x 21039529

[bibr54-19485506251411575] Paine A. E. Hill M. Rochester C. (2010). A rose by any other name. Revisiting the question: What exactly is volunteering? https://www.ifrc.org/docs/IDRL/Volunteers/a-rose-by-any-other-name-what-exactly-is-volunteering.pdf

[bibr55-19485506251411575] Pearce S. Kristjansson E. (2019). Physical and social perceptions of the neighbourhood and youth volunteerism: Canada’s capital region. Canadian Journal of Nonprofit and Social Economy Research, 10(1), 41–60. 10.22230/cjnser.2019v10n1a291

[bibr56-19485506251411575] Putnam R. D. (1993). What makes democracy work? National Civic Review, 82(2), 101–107. 10.1002/ncr.4100820204

[bibr57-19485506251411575] Putnam R. D. (2007). E Pluribus Unum: Diversity and community in the twenty-first century. Scandinavian Political Studies, 30(2), 137–174. 10.1111/j.1467-9477.2007.00176.x

[bibr58-19485506251411575] PytlikZillig L. M. Hamm J. A. Shockley E. Herian M. N. Neal T. M. S. Kimbrough C. D. Tomkins A. J. Bornstein B. H. (2016). The dimensionality of trust-relevant constructs in four institutional domains: Results from confirmatory factor analyses. Journal of Trust Research, 6(2), 111–150. 10.1080/21515581.2016.1151359

[bibr59-19485506251411575] PytlikZillig L. M. Kimbrough C. D. (2016). Consensus on conceptualizations and definitions of trust: Are we there yet? In Shockley E. Neal T. M. S. PytlikZillig L. M. Bornstein B. H. (Eds.), Interdisciplinary perspectives on trust: Towards theoretical and methodological integration (pp. 17–47). Springer. 10.1007/978-3-319-22261-5_2

[bibr60-19485506251411575] Rosseel Y. (2012). Lavaan: An R package for structural equation modeling. Journal of Statistical Software, 48(2), 1–36. http://www.jstatsoft.org/v48/i02/

[bibr61-19485506251411575] Rotolo T. Wilson J. (2011). State-level differences in volunteerism in the United States: Research based on demographic, institutional, and cultural macrolevel theories. Nonprofit and Voluntary Sector Quarterly, 41(3), 452–473. 10.1177/0899764011412383

[bibr62-19485506251411575] Sitkin S. B. Bijlsma-Frankema K. M. (2018). Distrust. In Searle R. Nienaber A.-M. Sitkin S. B. (Eds.), The Routledge companion to trust (pp. 50–61). Routledge.

[bibr63-19485506251411575] Sivesind K. H. Pospíšilová T. Frič P. (2013). Does volunteering cause trust? European Societies, 15(1), 106–130. 10.1080/14616696.2012.750732

[bibr64-19485506251411575] Six F. E. Latusek D. (2023). Distrust: A critical review exploring a universal distrust sequence. Journal of Trust Research, 13(1), 1–23. 10.1080/21515581.2023.2184376

[bibr65-19485506251411575] Snyder M. Omoto A. M. (2008). Volunteerism: Social issues perspectives and social policy implications. Social Issues and Policy Review, 2(1), 1–36. 10.1111/j.1751-2409.2008.00009.x

[bibr66-19485506251411575] Sønderskov K. M. (2011). Does generalized social trust lead to associational membership? Unravelling a bowl of well-tossed spaghetti. European Sociological Review, 27(4), 419–434. 10.1093/esr/jcq017

[bibr67-19485506251411575] Steenvoorden E. H. van der Meer T. W. G. (2021). National inspired or locally earned? The locus of local political support in a multilevel context. Frontiers in Political Science, 3, Article 642356. 10.3389/fpos.2021.642356

[bibr68-19485506251411575] Stürmer S. Snyder M. Kropp A. Siem B. (2006). Empathy-motivated helping: The moderating role of group membership. Personality and Social Psychology Bulletin, 32(7), 943–956. 10.1177/0146167206287363 16738027

[bibr69-19485506251411575] Stürmer S. Snyder M. Omoto A. M. (2005). Prosocial emotions and helping: The moderating role of group membership. Journal of Personality and Social Psychology, 88(3), 532–546. 10.1037/0022-3514.88.3.532 15740444

[bibr70-19485506251411575] Taniguchi H. (2013). The influence of generalized trust on volunteering in Japan. Nonprofit and Voluntary Sector Quarterly, 42(1), 127–147. 10.1177/0899764011434554

[bibr71-19485506251411575] Taniguchi H. Marshall G. A. (2014). The effects of social trust and institutional trust on formal volunteering and charitable giving in Japan. VOLUNTAS: International Journal of Voluntary and Nonprofit Organizations, 25(1), 150–175. 10.1007/s11266-012-9328-3

[bibr72-19485506251411575] Thomson M. (2002). Renewing volunteering for civic renewal. In Paxton W. Nash V. (Eds.), Any volunteers for the good society (pp. 17–27). Institute for Public Policy Research.

[bibr73-19485506251411575] UK Department for Culture Media & Sport. (2024). Community Life Survey 2023/24: Volunteering and charitable giving. UK Government. https://www.gov.uk/government/statistics/community-life-survey-202324-annual-publication/community-life-survey-202324-volunteering-and-charitable-giving

[bibr74-19485506251411575] Ullmann-Margalit E. (2004). Trust, distrust, and in between. In Hardin R. (Ed.), Distrust (pp. 60–82). Russell Sage Foundation.

[bibr75-19485506251411575] United Nations Volunteers. (2021). 2022 State of the World’s Volunteerism Report. Building equal and inclusive societies.

[bibr76-19485506251411575] van Ingen E. van der Meer T. (2016). Schools or pools of democracy? A longitudinal test of the relation between civic participation and political socialization. Political Behavior, 38(1), 83–103. 10.1007/s11109-015-9307-7

[bibr77-19485506251411575] Wilson J. (2000). Volunteering. Annual Review of Sociology, 26, 215–240. 10.1146/annurev.soc.26.1.215

[bibr78-19485506251411575] Wilson J. (2012). Volunteerism research: A review essay. Nonprofit and Voluntary Sector Quarterly, 41(2), 176–212. 10.1177/0899764011434558

[bibr79-19485506251411575] Wollebæk D. Strømsnes K. (2007). Voluntary associations, trust, and civic engagement: A multilevel approach. Nonprofit and Voluntary Sector Quarterly, 37(2), 249–263. 10.1177/0899764007304754

[bibr80-19485506251411575] Wong K. L. (2024). Overlapping functions: Volunteering and other forms of civic participation in the COVID-19 disaster. VOLUNTAS: International Journal of Voluntary and Nonprofit Organizations, 35(5), 854–865. 10.1007/s11266-024-00652-9

[bibr81-19485506251411575] Wu C. Wilkes R. (2017). Local–national political trust patterns: Why China is an exception. International Political Science Review, 39(4), 436–454. 10.1177/0192512116677587

[bibr82-19485506251411575] Yamada Y. Ćepulić D.-B. Coll-Martín T. Debove S. Gautreau G. Han H. Rasmussen J. Tran T. P. Travaglino G. A. Blackburn A. M. Boullu L. Bujić M. Byrne G. Caniëls M. C. J. Flis I. Kowal M. Rachev N. R. Reynoso-Alcántara V. Zerhouni O. . . .Consortium C. O. G. S. (2021). COVIDiSTRESS Global Survey dataset on psychological and behavioural consequences of the COVID-19 outbreak. Scientific Data, 8(1), 3. 10.1038/s41597-020-00784-9 33398078 PMC7782539

