# Supplementary material for: Does Political Trust Foster or Hinder Volunteering? A Longitudinal Investigation in the United Kingdom
Source: Soc Psychol Personal Sci. 2026 Jan 8;17(7):877–88. doi: 10.1177/19485506251411575 (PMC13375104; doi:10.1177/19485506251411575)
Supplement: sj-docx-1-spp-10.1177_19485506251411575 – Supplemental material for Does Political Trust Foster or Hinder Volunteering? A Longitudinal Investigation in the United Kingdom [file sj-docx-1-spp-10.1177_19485506251411575.docx]

**Does political trust foster or hinder volunteering? A longitudinal investigation in the UK**

**Supplementary Materials**

Table of Contents

[SM1. Full Demographic Profile of the Sample 2](#_Toc215746226)

[Table S1.1 Demographic of the full sample and subsamples who completed only two waves 2](#_Toc215746227)

[Table S1.2 Comparison of demographics between participants who returned vs. did not return for the subsequent wave 3](#_Toc215746228)

[SM2. Exploratory Factor Analysis on Participation Items 4](#_Toc215746229)

[Table S2.1 Results of the EFA on Participation Items at Wave 1 5](#_Toc215746230)

[Table S2.2 Results of the EFA on Participation Items at Wave 2 6](#_Toc215746231)

[Table S2.3 Results of the EFA on Participation Items at Wave 3 7](#_Toc215746232)

[SM3. Robustness Check: Analysis on Donating 8](#_Toc215746233)

[Table S3.1 Results from the Random-Intercept Cross-Lagged Model Testing the Associations between Trust in the National and Local Government and Donating 9](#_Toc215746234)

[Table S3.2 Results from the Random-Intercept Cross-Lagged Model Testing the Associations between Trust in the National and the Index of Volunteering-or-Donating 11](#_Toc215746235)

[SM4. The Role of Demographics on Trust and Volunteering 12](#_Toc215746236)

[Table S4.1 Correlation table 12](#_Toc215746237)

[Table S4.2 Linear regression analysis on trust in the national government at T1 12](#_Toc215746238)

[Table S4.3 Linear regression analysis on trust in the local government at T1 13](#_Toc215746239)

[Table S4.4 Logistic regression analysis on volunteering at T1 13](#_Toc215746240)

# **SM1.** Full Demographic Profile of the Sample

## **Table S1.1** Demographic of the full sample and subsamples who completed only two waves

|  | **Full sample** | | **Sample T1-T2** | | **Sample T2-T3** | |
| --- | --- | --- | --- | --- | --- | --- |
| **Demographic categories** | **Frequ.** | **%** | **Frequ.** | **%** | **Frequ.** | **%** |
| *Gender* |  |  |  |  |  |  |
| Male | 2216 | 44.0% | 1722 | 43.5% | 1592 | 42.9% |
| Female | 2805 | 55.7% | 2223 | 56.2% | 2108 | 56.8% |
| Other/Undisclosed | 18 | 0.3% | 12 | 0.3% | 14 | 0.3% |
| *Age* |  |  |  |  |  |  |
| 18-24 | 470 | 9.3% | 355 | 9.0% | 333 | 9.0% |
| 25-34 | 917 | 18.2% | 676 | 17.1% | 694 | 18.7% |
| 35-44 | 937 | 18.6% | 732 | 18.5% | 733 | 19.7% |
| 45-54 | 852 | 16.9% | 659 | 16.7% | 645 | 17.4% |
| 55-64 | 931 | 18.5% | 753 | 19.0% | 643 | 17.3% |
| 65-74 | 731 | 14.5% | 610 | 15.4% | 516 | 13.9% |
| 75+ | 201 | 4.0% | 172 | 4.3% | 150 | 4.0% |
| *Ethnicity* |  |  |  |  |  |  |
| White / White British | 4058 | 80.5% | 3104 | 78.4% | 3054 | 82.2% |
| Asian / Asian British | 407 | 8.1% | 366 | 9.3% | 281 | 7.6% |
| Black / African / Caribbean / Black British | 353 | 7.0% | 330 | 8.3% | 244 | 6.6% |
| Mixed / Multiple ethnicity | 90 | 1.8% | 70 | 1.8% | 64 | 1.7% |
| Other ethnicity | 59 | 1.2% | 45 | 1.1% | 36 | 1.0% |
| Undisclosed | 72 | 1.4% | 42 | 1.1% | 35 | 0.9% |
| *Socioeconomic status* |  |  |  |  |  |  |
| 8-rung Status ladder: *M* (*SD*) | 4.43 (1.25) | | 4.45 (1.25) | | 4.40 (1.25) | |
| *Political orientation* |  |  |  |  |  |  |
| Left-wing | 2307 | 45.8% | 1765 | 44.7% | 1829 | 49.2% |
| Center | 1559 | 31.0% | 1261 | 31.9% | 1052 | 28.3% |
| Right-wing | 1169 | 23.2% | 924 | 23.4% | 833 | 22.4% |
| Total | 5039 | 100% | 3957 | 100% | 3714 | 100% |

*Note*. Subjective socioeconomic status is measured on a ‘status ladder’ with 8 rungs numbered 1 to 8 (a higher number represents higher status). Political orientation is measured on a 7-point scale (1 = Left-wing, 4 = Centre, 7 = Right wing). As a subsample of participants filled all three waves (*n* = 2,632) and are thus included in the descriptive for both T1-T2 and T2-T3.

## **Table S1.2** Comparison of demographics between participants who returned vs. did not return for the subsequent wave

|  | **Wave 1** | | | **Wave 2** | | |
| --- | --- | --- | --- | --- | --- | --- |
| **Demographic** | **Returned to W2** | **Did not return** | **Test of difference** | **Returned to W3** | **Did not return** | **Test of difference** |
| Gender | 1722 M / 2222 F  (45.2% M) | 2028 M / 2528 F  (43.7% M) | χ^2^(1) = 1.88,  *p* = .17 | 1592 M / 2108 F  (43.6% M) | 2285 M / 2951 F  (43.0%) | χ^2^(1) = 0.31,  *p* = .58 |
| Age | 47.84 (16.68) | 47.73 (16.45) | *t*(8595) = 0.30,  *p* = .76 | 46.84 (16.41) | 44.1 (16.45) | *t*(8981) = 7.88,  *p* <.001 |
| Ethnicity | 810 NW / 3045 W  (17.4% NW) | 769 NW / 3642 W  (21.0% NW) | χ^2^(1) = 16.81,  *p* <.001 | 625 NW / 3054 W  (16.99% NW) | 1046 NW / 4167 W  (20.1% NW) | χ^2^(1) = 13.18,  *p* <.001 |
| Socioeconomic status | 4.46 (1.25) | 4.38 (1.34) | *t*(8348) = 2.81,  *p* = .005 | 4.40 (1.25) | 4.31 (1.35) | *t*(8705) = 3.19,  *p* = .001 |
| Political orientation | 3.56 (1.43) | 3.75 (1.47) | *t*(8577) = -6.17,  *p* <.001 | 3.45 (1.48) | 3.59 (1.54) | *t*(8981) = -4.59,  *p* <.001 |
| *N* | 3954 | 4643 | - | 3714 | 5269 | - |

*Note*. M = men, F = women, NW = non-White, W = White.

The relatively large attrition rates can be explained by the project’s methodology: while some participants were recruited through external providers (Prolific and Qualtrics Panel), many were recruited through advertising and snowball sampling via partnering civic society sector organisations and local authorities. Those are thus no “seasoned” respondents, and several simply declined the invitation to be recontacted for subsequent studies. Incentive for participation was also modest (£5, approx. $6.5, for a 30-min study). We believe this largely explains our attrition rates between waves (for more details, see Abrams, Broadwood, Lalot, Davies Hayon, et al., 2021).

# **SM2.** Exploratory Factor Analysis on Participation Items

As reported in the main text, the volunteering measure was extracted from a larger checklist that included different forms of political and civic action. Participants indicated in a dichotomous format (0 = no, 1 = yes) whether they had engaged in the activity during the past month. There are theoretical arguments why volunteering is conceptually different from other forms of action. Unlike many types of political participation or activism, volunteering is typically not intended to challenge or change the societal system. Rather, it accepts existing social structures and seeks to mitigate problems with this system without necessarily addressing their root causes (Wong, 2024). It is also more often carried out by wealthier or dominant groups in society, unlike typical minority-group-based collective action (Hustinx et al., 2022). As such, volunteerism and civic action are believed to arise from complementary processes in the civil sphere (Janoski, 2010).

However, we also wanted to make sure this distinction would show empirically. To this end, we conducted an exploratory factor analysis (EFA) on the 13 items of the civic action checklist measured at the first wave (we excluded the 14th option, “other”, given the low and heterogeneous answers to this item). We relied on a multiple criteria approach to determine the number of factors to extract. We considered the following methods: Ruscio’s comparison data, empirical Kaiser criterion, Hull’s method, and revised parallel analysis (see Auerswald & Moshagen, 2019). Most methods recommended extracting either one or three factors/dimensions (analysis conducted in R with the EFAtools package; Steiner & Grieder, 2020) :

- Comparison data: 1
- Empirical Kaiser criterion: 3
- Hull method with CAF: 1
- Hull method with CFI: 1
- Hull method with RMSEA: 1
- Parallel analysis with PCA: 3

The three-factor solution (WLSMV estimator to account for the binary nature of the data, oblimin rotation) provided a significantly better fit to the data than the single-factor solution, Δχ^2^(Δdf = 23) = 399.9, *p* < .001, and explained 58.0% of variance. Loadings are reported in Table S2.1. The analysis revealed that the item for volunteering (and, to a smaller extent, that of donating) loaded on a separate factor than most other actions.

## **Table S2.1** Results of the EFA on Participation Items at Wave 1

| **Item** | **Factor 1** | **Factor 2** | **Factor 3** | **Unique variance** | **Commonalities** |
| --- | --- | --- | --- | --- | --- |
| Signed a petition |  | .639 |  | .423 | .577 |
| Joined a political party | .412 |  | .312 | .433 | .567 |
| Written to an MP or councillor | .789 |  |  | .321 | .679 |
| Taken part in an online strike or demonstration |  | .570 |  | .452 | .548 |
| Decided not to buy or use particular products or services (boycott) |  | .588 |  | .564 | .436 |
| Tried to persuade people to support the cause |  | .585 |  | .499 | .501 |
| Showed posters or stickers to show your support |  | .540 |  | .466 | .534 |
| Supported a social media campaign (e.g. shared, liked or commented) |  | .921 |  | .288 | .712 |
| Opposed or criticised a social media campaign |  | .762 |  | .459 | .541 |
| Written to a newspaper | .742 |  |  | .397 | .603 |
| Endorsed children’s and young people’s protests |  | .664 |  | .394 | .606 |
| **Volunteered** |  |  | **.894** | .213 | .787 |
| **Made a donation** |  |  | **.483** | .556 | .444 |

*Note*. Loadings smaller than |.30| are not displayed.

This pattern replicated for the second wave of data collection (where the three-factor solution explained 56.5% of variance and provided significantly better fit than the single-factor solution, Δχ^2^(Δdf = 25) = 722.2, *p* < .001; Table S2.2) and the third one (47.5% variance explained, model comparison: Δχ^2^(Δdf = 27) = 366.7, *p* < .001; Table S2.3).

## **Table S2.2** Results of the EFA on Participation Items at Wave 2

| **Item** | **Factor 1** | **Factor 2** | **Factor 3** | **Unique variance** | **Commonalities** |
| --- | --- | --- | --- | --- | --- |
| Signed a petition | .579 | .355 |  | .381 | .619 |
| Joined a political party | .408 |  |  | .513 | .487 |
| Written to an MP or councillor | .758 |  |  | .398 | .602 |
| Taken part in an online strike or demonstration |  | .740 |  | .384 | .616 |
| Decided not to buy or use particular products or services (boycott) |  | .489 |  | .559 | .441 |
| Tried to persuade people to support the cause |  | .602 |  | .504 | .496 |
| Showed posters or stickers to show your support |  | .597 |  | .503 | .497 |
| Supported a social media campaign (e.g. shared, liked or commented) |  | .831 |  | .310 | .690 |
| Opposed or criticised a social media campaign |  | .833 |  | .372 | .628 |
| Written to a newspaper | .559 |  |  | .623 | .377 |
| Endorsed children’s and young people’s protests |  | .644 |  | .419 | .581 |
| **Volunteered "formally" (through an official charity or organisation)** |  |  | **.764** | .297 | .703 |
| **Helping out "informally" (personally or through an informal organisation such as mutual aid group)** |  |  | **.810** | .345 | .655 |
| **Made a donation** |  |  | **.531** | .477 | .523 |

*Note*. Loadings smaller than |.30| are not displayed.

## **Table S2.3** Results of the EFA on Participation Items at Wave 3

| **Item** | **Factor 1** | **Factor 2** | **Factor 3** | **Unique variance** | **Commonalities** |
| --- | --- | --- | --- | --- | --- |
| Signed a petition | .923 |  |  | .075 | .925 |
| Joined a political party |  | .561 |  | .547 | .453 |
| Written to an MP or councillor | .339 |  |  | .618 | .382 |
| Taken part in an online strike or demonstration |  | .740 |  | .414 | .586 |
| Taken part in a strike or demonstration in person |  | .798 |  | .345 | .655 |
| Decided not to buy or use particular products or services (boycott) |  | .465 |  | .561 | .439 |
| Tried to persuade people to support the cause |  | .638 |  | .468 | .532 |
| Showed posters or stickers to show your support |  | .644 |  | .402 | .598 |
| Supported a social media campaign (e.g. shared, liked or commented) |  | .675 |  | .410 | .590 |
| Opposed or criticised a social media campaign |  | .878 |  | .347 | .653 |
| Written to a newspaper |  | .436 |  | .735 | .265 |
| Endorsed children’s and young people’s protests |  | .781 |  | .341 | .659 |
| **Volunteered "formally" (through an official charity or organisation)** |  |  | **.644** | .373 | .627 |
| **Helping out "informally" (personally or through an informal organisation such as mutual aid group)** |  |  | **.765** | .408 | .592 |
| **Made a donation** |  |  | **.613** | .426 | .574 |

*Note*. Loadings smaller than |.30| are not displayed.

# **SM3.** Robustness Check: Analysis on Donating

As a robustness check, we investigated the relationships between trust and donating (single item) as well as a single indicator of donating-or-volunteering. The results of these supplemental analyses are reported here.

**Analysis on Donating (Single-Item)**

Across waves, respectively 32.7%, 23.6%, and 21.6% of respondents reported having made a donation during the past month. We conducted a similar RI-CLPM (same strategy as described in the main text) on this single indicator. The model showed excellent fit to the data, χ^2^ = 177.89, df = 22, χ^2^/df = 8.09, CFI = .983, RMSEA = .038, 90% CI [.032, .043], SRMR = .016. Results are detailed in Table S3.1.

The most important findings from the within-person regressions were replicated: national trust had a lagged negative effect on the likelihood to donate three months later (β_T1/T2_ = -.243 / -.299, *p* = .002; marginal effect = -.132 or decrease of 13.2 percentage points by 1-unit increase in national trust), while local trust had a positive effect (β = .13, *p* = .042; marginal effect = .069). Donating showed greater stability over time than volunteering did.

## **Table S3.1** Results from the Random-Intercept Cross-Lagged Model Testing the Associations between Trust in the National and Local Government and Donating

| **Association** | **Est.** | ***SE*** | **95% CI** | ***z*-test** | ***p*-value** | **β (T1 / T2)** |
| --- | --- | --- | --- | --- | --- | --- |
| **Between-person covariances (random intercepts)** |  |  |  |  |  |  |
| National trust ~~ Local trust | .266 | .028 | [.210, .321] | 9.42 | <.001 | .339 |
| National trust ~~ Donating | -.032 | .098 | [-.223, .160] | -0.32 | .75 | -.017 |
| Local trust ~~ Donating | .135 | .067 | [.003, .267] | 2.01 | .045 | .096 |
| **Within-person covariances at T1** |  |  |  |  |  |  |
| National trust ~~ Local trust | .131 | .027 | [.079, .183] | 4.94 | <.001 | .273 |
| National trust ~~ Donating | -.344 | .122 | [-.582, -.105] | -2.82 | .005 | -.212 |
| Local trust ~~ Donating | .089 | .088 | [-.084, .262] | 1.01 | .31 | .052 |
| **Within-person covariances at T2 & T3** |  |  |  |  |  |  |
| National trust ~~ Local Trust | .122 | .016 | [.091, .153] | 7.65 | <.001 | .274 |
| National trust ~~ Donating | -.054 | .043 | [-.139, .031] | -1.25 | .21 | -.078 |
| Local trust ~~ Donating | .014 | .038 | [-.061, .089] | 0.38 | .71 | .022 |
| **Within-person regressions at T2 & T3** |  |  |  |  |  |  |
| National trust_T_ |  |  |  |  |  |  |
| ~ National trust_T-1_ | .391 | .068 | [.257, .525] | 5.72 | <.001 | .346 / .390 |
| ~ Local trust_T-1_ | -.106 | .037 | [-.178, -.033] | -2.84 | .004 | -.099 / -.091 |
| ~ Donating_T-1_ | -.055 | .016 | [-.086, -.024] | -3.45 | .001 | -.174 / -.088 |
| Local trust_T_ |  |  |  |  |  |  |
| ~ National trust_T-1_ | -.072 | .041 | [-.152, .008] | -1.77 | .076 | -.074 / -.084 |
| ~ Local trust_T-1_ | .151 | .041 | [.071, .232] | 3.69 | <.001 | .165 / .152 |
| ~ Donating_T-1_ | .024 | .015 | [-.005, .053] | 1.61 | .11 | .087 / .044 |
| Donating_T_ |  |  |  |  |  |  |
| ~ National trust_T-1_ | -.439 | .139 | [-.710, -.167] | -3.17 | .002 | -.243 / -.299 |
| ~ Local trust_T-1_ | .227 | .112 | [.008, .446] | 2.03 | .042 | .133 / .133 |
| ~ Donating_T-1_ | .233 | .047 | [.141, .324] | 4.99 | <.001 | .460 / .253 |

*Note*. Covariances are indicated as x_1_ ~~ x_2_. Regressions are indicated as y ~ x (i.e., the fully left justified variable is the endogenous variable). Within-person covariances at Time 1 reflect the relationship between the variables after accounting for their stable, trait-like individual differences, but not influenced by prior time points (since it is the first measurement). Within-person covariances at Times 2-3 represent the residual (state-like) associations, controlling for both stable, trait-like differences (random intercepts), and previous within-person fluctuations due to the cross-lagged and autoregressive processes. It should be noted that for binary outcomes (here, donating), standardised coefficients are defined on the underlying latent response variable and should not be interpreted directly as standardised effects on the observed binary variable. Although the point estimate is constrained to equality, the effects are standardised based on the point estimate and variances; and since the variances vary at each assessment occasion, the standardised effects are slightly different at T2 and T3.

**Analysis on Donating or Volunteering**

We finally assessed a single indicator showing whether respondents had *either* volunteered or made a donation during the past month (=1) or not (=0). Across waves, respectively 38.2%, 28.2%, and 26.7% of respondents reported either having made a donation or volunteering during the past month.

The RI-CLPM showed excellent fit to the data, χ^2^ = 188.29, df = 22, χ^2^/df = 8.56, CFI = .982, RMSEA = .039, 90% CI [.034, .044], SRMR = .018. Results are detailed in Table S3.2. Again, the most important findings from the within-person regressions were replicated: national trust had a lagged negative effect on the likelihood to donate three months later (β_T1/T2_ = -.309 / -.377, *p* < .001; marginal effect = -.199 or decrease of 19.9 percentage points by 1-unit increase in national trust), while local trust had a positive effect (β_T1/T2_ =.210 / .209, *p* < .001; marginal effect = .125). This index also showed good stability over time.

## **Table S3.2** Results from the Random-Intercept Cross-Lagged Model Testing the Associations between Trust in the National and the Index of Volunteering-or-Donating

| **Association** | **Est.** | ***SE*** | **95% CI** | ***z*-test** | ***p*-value** | **β (T1 / T2)** |
| --- | --- | --- | --- | --- | --- | --- |
| **Between-person covariances (random intercepts)** |  |  |  |  |  |  |
| National trust ~~ Local trust | .276 | .029 | [.220, .333] | 9.56 | <.001 | .355 |
| National trust ~~ Don/Vol. | .047 | .103 | [-.154, .248] | 0.46 | .64 | .025 |
| Local trust ~~ Don/Vol. | .122 | .072 | [-.018, .263] | 1.70 | .089 | .086 |
| **Within-person covariances at T1** |  |  |  |  |  |  |
| National trust ~~ Local trust | .122 | .027 | [.068, .175] | 4.46 | <.001 | .253 |
| National trust ~~ Don/Vol. | -.354 | .130 | [-.609, -.098] | -2.72 | .007 | -.207 |
| Local trust ~~ Don/Vol. | .176 | .094 | [-.007, .360] | 1.88 | .060 | .096 |
| **Within-person covariances at T2 & T3** |  |  |  |  |  |  |
| National trust ~~ Local Trust | .124 | .016 | [.093, .156] | 7.75 | <.001 | .282 |
| National trust ~~ Don/Vol. | -.079 | .041 | [-.160, .002] | -1.90 | .057 | -.115 |
| Local trust ~~ Don/Vol. | -.006 | .037 | [-.079, .067] | -0.16 | .87 | -.009 |
| **Within-person regressions at T2 & T3** |  |  |  |  |  |  |
| National trust_T_ |  |  |  |  |  |  |
| ~ National trust_T-1_ | .372 | .075 | [.226, .519] | 5.00 | <.001 | .330 / .373 |
| ~ Local trust_T-1_ | -.103 | .038 | [-.177, -.028] | -2.70 | .007 | -.098 / -.091 |
| ~ Don/Vol._T-1_ | -.062 | .015 | [-.092, -.032] | -4.10 | <.001 | -.210 / -.106 |
| Local trust_T_ |  |  |  |  |  |  |
| ~ National trust_T-1_ | -.090 | .041 | [-.170, -.009] | -2.19 | .029 | -.090 / -.103 |
| ~ Local trust_T-1_ | .173 | .042 | [.091, .254] | 4.15 | <.001 | .187 / .174 |
| ~ Don/Vol._T-1_ | .036 | .014 | [.009, .063] | 2.64 | .008 | .139 / .071 |
| Don/Vol._T_ |  |  |  |  |  |  |
| ~ National trust_T-1_ | -.594 | .135 | [-.860, -.329] | -4.39 | <.001 | -.309 / -.377 |
| ~ Local trust_T-1_ | .374 | .110 | [.158, .589] | 3.40 | .001 | .210 / .209 |
| ~ Don/Vol._T-1_ | .230 | .042 | [.148, .312] | 5.51 | <.001 | .458 / .248 |

*Note*. Covariances are indicated as x_1_ ~~ x_2_. Regressions are indicated as y ~ x (i.e., the fully left justified variable is the endogenous variable). Within-person covariances at Time 1 reflect the relationship between the variables after accounting for their stable, trait-like individual differences, but not influenced by prior time points (since it is the first measurement). Within-person covariances at Times 2-3 represent the residual (state-like) associations, controlling for both stable, trait-like differences (random intercepts), and previous within-person fluctuations due to the cross-lagged and autoregressive processes. It should be noted that for binary outcomes (here, donating), standardised coefficients are defined on the underlying latent response variable and should not be interpreted directly as standardised effects on the observed binary variable. Although the point estimate is constrained to equality, the effects are standardised based on the point estimate and variances; and since the variances vary at each assessment occasion, the standardised effects are slightly different at T2 and T3.

# **SM4.** The Role of Demographics on Trust and Volunteering

Here we report more detail regarding the role of demographics on trust and volunteering. Table S4.1 shows the correlations between demographics (gender, age, ethnicity, socioeconomic status, and political orientation) and the variables of interest of the study. Tables S4.2 to S4.4 report the results of linear regression analysis regressing trust in the national government (S4.2), trust in the local government (S4.3), and volunteering measured at T1 (S4.4; logistic regression) on demographics.

## **Table S4.1** Correlation table

|  | **National trust** | | | **Local trust** | | | **Volunteering** | | |
| --- | --- | --- | --- | --- | --- | --- | --- | --- | --- |
|  | **T1** | **T2** | **T3** | **T1** | **T2** | **T3** | **T1** | **T2** | **T3** |
| Gender (Woman) | -.04* | -.03 | -.05* | .00 | .03 | .01 | .04* | .03 | .03 |
| Age | .15*** | .19*** | .14*** | .16*** | .16*** | .15*** | .05** | .08*** | .06** |
| Ethnicity (White) | .08*** | .05*** | .00 | .08*** | .08*** | .05** | -.02 | .03 | .01 |
| SES | .06*** | .08*** | .10*** | .12*** | .11*** | .12*** | .10*** | .08*** | .08*** |
| Political orientation | .45*** | .50*** | .48*** | -.03 | .00 | -.04* | -.08*** | -.09*** | -.09*** |

*Note*. Gender is recoded -1 = men, +1 = women. Ethnicity is recoded -1 = other than White, 1 = White. SES = subjective socioeconomic status (status ladder). T1-T2-T3 = time of measurement 1, 2, 3. * *p* <.05, ** *p* <.01, *** *p* <.001.

## **Table S4.2** Linear regression analysis on trust in the national government at T1

|  | ***b*** | ***SE*** | **95% CI** | ***t*-test** | ***p*-value** | **β** |
| --- | --- | --- | --- | --- | --- | --- |
| Intercept | .755 | .084 | [.590, .921] | 8.95 | <.001 | - |
| Gender (Woman) | .044 | .018 | [.008, .080] | 2.41 | .016 | .036 |
| Age | .003 | .001 | [.001, .006] | 2.88 | .004 | .047 |
| Ethnicity (White) | .039 | .024 | [-.007, .085] | 1.66 | .098 | .026 |
| SES | -.001 | .015 | [-.029, .028] | -0.04 | .97 | -.001 |
| Political orientation | .375 | .013 | [.350, .401] | 29.28 | <.001 | .437 |

*Note*. Gender is recoded -1 = men, +1 = women. Ethnicity is recoded -1 = other than White, 1 = White. SES = subjective socioeconomic status (status ladder).

## **Table S4.3** Linear regression analysis on trust in the local government at T1

|  | ***b*** | ***SE*** | **95% CI** | ***t*-test** | ***p*-value** | **β** |
| --- | --- | --- | --- | --- | --- | --- |
| Intercept | 2.444 | .079 | [2.291, 2.603] | 30.81 | <.001 | - |
| Gender (Woman) | .024 | .017 | [-.010, .058] | 1.41 | .16 | .023 |
| Age | .009 | .001 | [.007, .011] | 8.13 | <.001 | .146 |
| Ethnicity (White) | .039 | .022 | [-.005, .082] | 1.77 | .077 | .030 |
| SES | .084 | .014 | [.056, .111] | 6.07 | <.001 | .100 |
| Political orientation | -.048 | .012 | [-.071, -.024] | -3.98 | <.001 | -.065 |

*Note*. Gender is recoded -1 = men, +1 = women. Ethnicity is recoded -1 = other than White, 1 = White. SES = subjective socioeconomic status (status ladder).

## **Table S4.4** Logistic regression analysis on volunteering at T1

|  | ***Est.*** | ***SE*** | ***z*-test** | ***p*-value** | **OR** | **OR 95% CI** |
| --- | --- | --- | --- | --- | --- | --- |
| Intercept | -2.67 | .223 | -11.97 | <.001 | 0.07 | [0.04, 0.11] |
| Gender (Woman) | .149 | .047 | 3.20 | .001 | 1.16 | [1.06, 1.27] |
| Age | .012 | .003 | 3.91 | <.001 | 1.01 | [1.01, 1.02] |
| Ethnicity (White) | -.137 | .058 | -2.35 | .019 | 0.87 | [0.78, 0.98] |
| SES | .236 | .038 | 6.17 | <.001 | 1.27 | [1.18, 1.37] |
| Political orientation | -.166 | .032 | -5.15 | <.001 | 0.85 | [0.80, 0.90] |

*Note*. Gender is recoded -1 = men, +1 = women. Ethnicity is recoded -1 = other than White, 1 = White. SES = subjective socioeconomic status (status ladder). Volunteering is recoded 0 = did not volunteer, 1 = volunteered. OR = Odd Ratio.

**Supplementary References**

Auerswald, M., & Moshagen, M. (2019). How to determine the number of factors to retain in exploratory factor analysis: A comparison of extraction methods under realistic conditions. *Psychological Methods*, *24*(4), 468-491. <https://doi.org/10.1037/met0000200>

Steiner, M. D., & Grieder, S. (2020). EFAtools: An R package with fast and flexible implementations of exploratory factor analysis tools. *Journal of Open Source Software*, *5*(53), 2521. <https://doi.org/10.21105/joss.02521>
